# Rapid Release of Tissue Enzymes into Blood after Blast Exposure: Potential Use as Biological Dosimeters

**DOI:** 10.1371/journal.pone.0033798

**Published:** 2012-04-06

**Authors:** Peethambaran Arun, Samuel Oguntayo, Yonas Alamneh, Cary Honnold, Ying Wang, Manojkumar Valiyaveettil, Joseph B. Long, Madhusoodana P. Nambiar

**Affiliations:** 1 Blast-Induced Neurotrauma Branch, Center for Military Psychiatry and Neuroscience, Walter Reed Army Institute of Research, Silver Spring, Maryland, United States of America; 2 Veterinary Services Program, Division of Pathology, Walter Reed Army Institute of Research, Silver Spring, Maryland, United States of America; University of South Florida, United States of America

## Abstract

Explosive blast results in multiple organ injury and polytrauma, the intensity of which varies with the nature of the exposure, orientation, environment and individual resilience. Blast overpressure alone may not precisely indicate the level of body or brain injury after blast exposure. Assessment of the extent of body injury after blast exposure is important, since polytrauma and systemic factors significantly contribute to blast-induced traumatic brain injury. We evaluated the activity of plasma enzymes including aspartate aminotransferase (AST), alanine aminotransferase (ALT), lactate dehydrogenase (LDH) and creatine kinase (CK) at different time points after blast exposure using a mouse model of single and repeated blast exposures to assess the severity of injury. Our data show that activities of all the enzymes in the plasma were significantly increased as early as 1 h after blast exposure. The elevated enzyme activity remained up to 6 h in an overpressure dose-dependent manner and returned close to normal levels at 24 h. Head-only blast exposure with body protection showed no increase in the enzyme activities suggesting that brain injury alone does not contribute to the systemic increase. In contrast to plasma increase, AST, ALT and LDH activity in the liver and CK in the skeletal muscle showed drastic decrease at 6 h after blast exposures. Histopathology showed mild necrosis at 6 h and severe necrosis at 24 h after blast exposures in liver and no changes in the skeletal muscle suggesting that the enzyme release from the tissue to plasma is probably triggered by transient cell membrane disruption from shockwave and not due to necrosis. Overpressure dependent transient release of tissue enzymes and elevation in the plasma after blast exposure suggest that elevated enzyme activities in the blood can be potentially used as a biological dosimeter to assess the severity of blast injury.

## Introduction

The frequency of blast-induced traumatic brain injury (blast TBI) has been increased tremendously in the recent conflicts due to the high use of improvised explosive devises [1], [2]. Although the precise cause and mechanisms of blast TBI remains unclear, blast TBI shares clinical features of both penetrating TBI and closed-head TBI [3]. The uniqueness of blast TBI compared to other types of TBI is concurrent organ injury and polytrauma due to the whole body exposure to blast. Hemorrhage, inflammation and oxidative stress after blast exposure are not only confined to brain but also occur in other gas filled body organs such as lungs, gastrointestinal tracts and auditory systems [4]–[8].

The neuropathology and subsequent cognitive deficits after blast exposure are proposed to be a cumulative effect of direct blast overpressure effect on the brain along with damage to other body organs [9]–[11]. Protective Kevlar body vests decreased the mortality, neuropathology and behavioral deficits in rats exposed to blast overpressure supporting the notion that polytrauma and systemic effects significantly contribute to blast TBI [12]. Recently it has been reported that blast exposure to torso after head protection produce more severe neurotrauma compared to head-only exposure supporting that body injury, polytrauma and systemic factors play a significant role in blast TBI [13], [14]. Furthermore, torso protection but not head protection significantly attenuated blast neurotrauma indicating that blast mediated organ injury, polytrauma and systemic response plays a vital role in the development of primary blast neurotrauma [13], [14]. Systemic response along with transient torso to brain hydraulic overpressure transmission leads to blood-brain barrier breakdown, neuroinflammation, cerebral vasospasm and ultimately blast neurotrauma [3], [13], [15]–[21].

One of the drawbacks in the identification as well as timely treatment of blast TBI is the difficulty in early diagnosis of the extent of brain and body injury to blast in the absence of any physical injury in most cases. Also, the threshold of blast overpressure for TBI is not well understood and not uniform among the population. Moreover, the nature of exposure, orientation of the subject and surrounding environment plays decisive roles in blast TBI or body injury [22]. Recently, a colorimetric blast injury dosimeter which can detect high blast overpressure exposure by changing the color and ultrastructure of the photonic crystalline material in an overpressure dependant manner has been reported [23]. Blast overpressure measurement alone may not exactly reflect the extent of body injury or blast TBI. Early determination of the extent of body exposure to blast not only enables assessing the severity of injury but also helps providing appropriate medical care and prevent the victims from immediately returning to the duty which can further exacerbate the injury with more exposures or other co-morbidity factors [23].

Organ specific proteins/enzymes as well as microRNAs have been reported to be secreted in to the blood after an insult and are being studied as specific biomarkers of injury including TBI [24]–[28]. Significant amount of proteins which are abundant in the brain including glial fibrillary acidic protein, neuron-specific enolase and S100B have been found to be present in the blood after injury to the brain [27], [28]. The release of these proteins/enzymes to blood requires blood-brain barrier breakdown after injury. Proteins/enzymes abundant in liver, kidney and heart are also found to be elevated in the blood circulation after injury to the respective organs [29]–[31]. Aspartate aminotransferase (AST), alanine aminotransferase (ALT) and lactate dehydrogenase (LDH) are highly expressed in the liver and their elevated levels in blood circulation have been used for the diagnosis and prognosis of liver damage [32], [33]. Similarly, the levels of creatine kinase (CK), which is abundant in the skeletal muscle, increase significantly in the plasma after damage to muscle [34], [35]. Using an *in vitro* blast TBI model system with shock tube, we have shown that blast exposure leads to the release of LDH from the cells to the culture medium without cell mortality and suggested that blast-induced plasma membrane damage as a potential mechanism of this enzyme release [36]. We considered that activities of enzymes can increase in the blood if there is any type of injury to the organs, including cell membrane rupture due to shockwave after blast exposures and may reflect the severity of injury and the intensity of exposure.

In the present study, by using a mice model of single and repeated blast exposures, we measured the activities of AST, ALT, LDH and CK in the plasma at different time intervals after blast exposures for potential application as biological dosimeters of blast injury.

## Results

### Effect of blast overpressure dose on plasma enzyme activity

**Figure 1:**
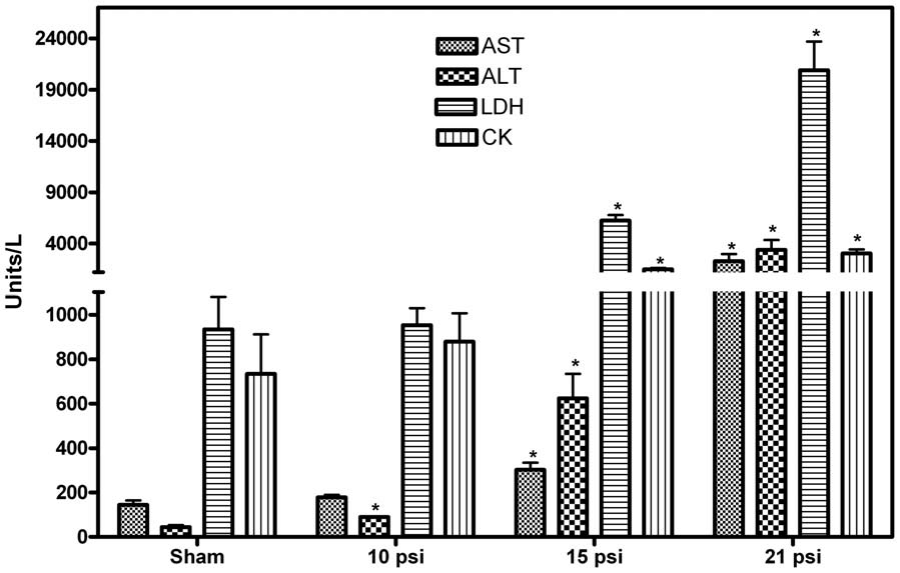
Blast overpressure dose response on the activities of enzymes in the plasma after 1 h. The activities of AST, ALT, LDH and CK increases in the plasma in an overpressure dose dependant manner. Mean ± SEM values of enzyme activities after exposure to different levels of blast overpressures are compared to sham. Statistical analysis was carried out by ANOVA test (n = 6). * *p*<0.05.

Increase in plasma enzyme activities after different levels of blast overpressure exposure is shown in Fig. 1. Plasma enzyme activity increase was directly proportional to the blast overpressure levels. At 10 psi overpressure, except ALT, none of the enzyme activities measured were increased significantly at 1 h after single blast exposure suggesting that ALT is the most sensitive enzyme for blast exposure. All the enzymes studied showed significant increase in the plasma at 1 h after exposure to 15 and 21 psi blast overpressures.

### Enzyme activities in the plasma after single, repeated and head-only blast exposures

Table 1 shows the activities of AST, ALT, LDH and CK respectively at 1, 6 and 24 h in the plasma of mice exposed to single and repeated blasts at 21 psi with and without protective vest. All the enzymes studied in the plasma showed a transient increase as early as 1 h and remained elevated at least up to 6 h post-blast. Compared to single blast exposure, repeated exposures did not further increase the enzyme activities at 6 h (Table 1). The activities of LDH and CK in the plasma of blast exposed mice returned to normal levels by 24 h post-blast exposures, whereas the activities of AST and ALT were still significantly elevated compared to respective sham controls. The highest increase was observed in the activity of ALT and was approximately 131-fold at 6 h after single blast exposure. The activities of LDH and AST showed approximately 34 and 20-fold increase respectively at 6 h after repeated blast exposures. Activity of CK showed a maximum of 6-fold increase in the plasma at 1 h after repeated blast exposures. Repeated blast exposure of animals with protective vest covering the whole body except the head completely prevented the increase of enzyme activities in the plasma (Table 1).

**Table 1:** Enzyme activities in the plasma at different time intervals after single and repeated blast exposures at 21 psi with and without protective vest.

	AST (Units/L)	ALT (Units/L)	LDH (Units/L)	CK (Units/L)
Sham	144.57±52.86	44.14±22.52	935.14±384.36	734.00±469.38
1 blast, 6 h, no vest	2739.33±1281.63*	5786.00±3845.01*	31776.33±24597.79*	2318.33±894.89*
3 blasts, 1 h, no vest	2708.33±1490.17*	3924.33±2049.52*	26399.67±8146.89*	4765.66±27.37*
3 blasts, 6 h, no vest	2940.66±1075.15*	5216.50±2165.76*	32135.17±4153.18*	1855.83±968.52*
3 blasts, 6 h, with vest	147.20±63.40	91.00±44.23	978.80±333.81	676.80±267.65
3 blasts, 24 h, no vest	392.80±156.25*	1133.40±1010.11*	2627.60±2307.97	548.20±270.07

Mean ± SEM values of different blast exposure groups were compared to those of sham. AST, aspartate aminotransferase; ALT, alanine aminotransferase; LDH, lactate dehydrogenase; CK, creatine kinase; Statistical analysis was carried out by ANOVA test (n = 7).

* p<0.05.

### Effect of blast exposure on activities of enzymes in the tissue

The enzymes AST, ALT and LDH are abundant in the liver. The activities of these enzymes in the liver were determined at 6 h post-blast (21 psi), the time period at which their activity was maximum in the plasma. The activity of CK was determined at 6 h after blast exposure in the skeletal muscle, a tissue which is rich with this enzyme. The results in Table 2 show that the activities of these enzyme decreased significantly in the tissues after blast exposure. At 24 h, the activities of the enzymes were higher in the tissues compared to 6 h after repeated blast exposures.

**Table 2:** Enzyme activities in the liver and muscle tissues at 6 and 24 h after repeated blast exposures.

Tissue	Enzyme	Sham	6 h post-blast	24 h post-blast
Liver	AST (Units/mg protein)	4.70±1.29	1.28±0.70*	2.68±0.97*
Liver	ALT (Units/mg protein)	7.41±3.27	0.89±0.65*	3.47±1.02*
Liver	LDH (Units/mg protein)	38.01±18.62	0.56±0.43*	12.59±3.87*
Muscle	CK (Units/µg protein)	101.03±16.62	50.55±18.99*	71.48±12.57*

Mean ± SEM values at each time points are compared to those of sham. AST, aspartate aminotransferase; ALT, alanine aminotransferase; LDH, lactate dehydrogenase; CK, creatine kinase. Statistical analysis was carried out by ANOVA test (n = 7).

* p<0.05.

### Histopathology of the liver and skeletal muscle after blast exposure

**Figure 2:**
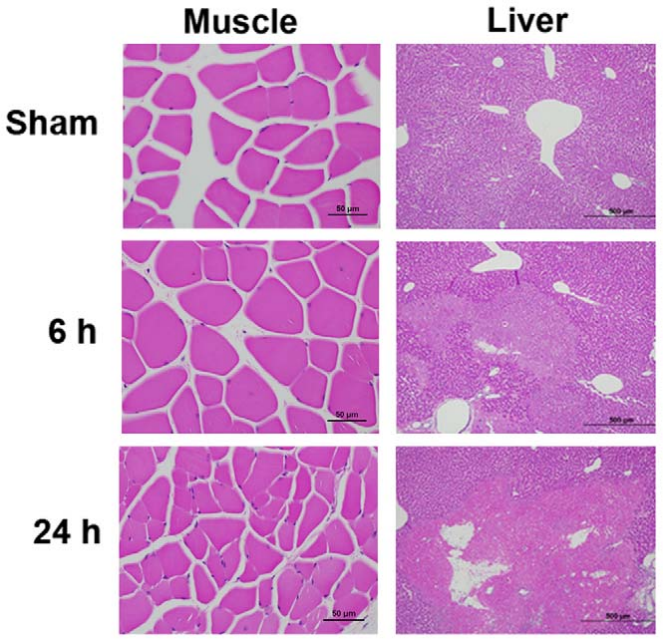
Hematoxylin and Eosin staining of liver and muscle at different time intervals after blast exposure. In the liver of blast exposed mice, the area with maximum lesion was showed. 6 h post-blast exposure showed the initiation of necrosis with mild discoloration of a large area of the tissue, whereas after 24 h, there is more widespread necrosis with increased neutrophilic infiltration resulting in significant tissue damage and stromal collapse. Liver −10× (500 µm) and muscle −40× (50 µm) magnifications.

Histopathology of liver and skeletal muscle at 6 and 24 h in the mice exposed to repeated blasts is shown in Fig. 2. No histopathological changes were observed in the skeletal muscle at both the time points studied. On the other hand, in the case of liver, there were foci of moderate coagulative necrosis with pyknosis, karyolysis, or nuclear absence randomly and multifocally at 6 h time point after exposure. At 24 h post-blast, the histopathological changes of liver were more severe with significant neutrophil infiltration.

## Discussion

In this study, we demonstrated for the first time, a transient increase in the activities of tissue enzymes in the plasma after single and repeated blast exposures. The enzymes activities of AST, ALT and LDH which are highly abundant in the liver were found to be increased several folds in the blood circulation as early as 1 h after blast exposure (Table 1). The activity of all the enzymes remained elevated in the plasma at least up to 6 h post-blast exposure. The activities of ALT and AST also showed statistically significant elevation in the plasma at 24 h. Plasma LDH activity reached to almost normal levels by 24 h post-blast. The decrease in the activities of enzymes in the plasma at 24 h compared to 6 h suggests possible rapid elimination by the renal clearance system probably due to their lower molecular weights.

The significant increase in the activities of all the three liver enzymes in the plasma at 6 h post-blast exposure was associated with a simultaneous decrease in the liver tissue (Table 2) suggesting that they are released rapidly from the liver after blast exposure. The mechanism of enzyme release seems to be a consequence of cell membrane disruption. Instantaneous cellular pressure rise and drop after blast exposure may be a factor responsible for the disruption of cell membrane. Histopathological examination of liver showed severe necrosis at 24 h post-blast whereas only mild necrosis at 6 h (Fig. 2) indicating that the release of liver enzymes is independent on blast-induced necrosis of liver and further support the hypothesis of cell membrane disruption after blast exposure as a mechanism of transient enzyme release. Moreover, the activities of the enzymes in the liver tissue at 24 h was significantly higher compared to 6 h post-blast exposures suggesting that there is no positive correlation between the severity of liver damage/necrosis and plasma or liver enzyme activities at 6 or 24 h. However, the initial increase in the blood enzyme activity may predict the severity of eventual liver damage after blast.

Similar results were observed with CK activity in the plasma and skeletal muscle (Tables 1 and 2). Histopathological analysis of skeletal muscle showed no any significant changes at 6 or 24 h after repeated blast exposures (Fig. 2) which support the possible release of CK from skeletal muscle through transient membrane rupture after blast exposure and rules out the possibility of muscle fiber necrosis leading to release of CK.

In the mice model of tightly coupled repetitive blast-induced TBI, the neurobiological effects of blast exposure increased significantly with the number of exposures [37]. However, the increase in plasma enzyme activities was similar between single or repeated blast exposures suggesting that maximum enzyme release occurs after single blast and multiple blasts do not lead to further increase in the plasma enzyme activities. The reason for the lack of cumulative effects is not clear, but these results implicate that systemic factors contributing to TBI may be similar between single or repeated blasts and increase in TBI after repeated blasts probably involves more direct effects of blast exposure to the brain. Also, different organs may have differences in vulnerability to repeated blast exposures.

Brain water content or edema was significantly higher as early as 4 h after repetitive blast exposures where as no significant difference was observed at 24 h post-blast exposures compared to sham controls [37] suggesting possible transient neuronal membrane rupture leading to rapid movement of water molecules into the cells. In rats exposed to underwater shockwaves using a micro explosion devise, leakage of administered Evans blue dye in and around the area of lesion has been reported indicating increase in cell membrane permeability as an impending mechanism of brain edema [21]. The view of abrupt membrane rupture is also supported by our earlier studies showing potential plasma membrane disruption and release of LDH from cell lines to extracellular medium after blast exposure without cell mortality [36]. Shockwaves have been utilized as a method for increasing the cell permeability to introduce macromolecules and small polar molecules into the cells for gene therapy and anticancer drug delivery [38]–[42]. Shockwave exposure was found to increase the permeability of leukemia cells and it has been reported that the shockwaves rather than the overpressure increases membrane permeability without any significant cell mortality [38].

Significant increase in the incidence of blast TBI in the current wars urged the need to determine blast thresholds that can induce TBI. Recently a material-based colorimetric blast injury dosimeter has been reported [23]. The blast overpressures used in that study (59 to 158 psi) were significantly higher than that was used in our experiments (10 to 21 psi) suggesting that measurement of plasma enzyme activity is a more sensitive method. Significant elevation of all the four enzymes studied in the plasma by 1 h after 15 psi blast overpressure exposure (Fig. 1), suggest that the increases in the activities of these enzymes in the plasma can be used as a biological dosimeter of the severity of blast exposure. Increase in the enzyme activity is a direct measure of the extent of blast-induced body injury. It might also represent the severity of TBI, since more severe body injury release higher levels of enzymes and systemic factors after single blast exposure and exacerbate brain injury [13], [14]. Since the basal levels of these enzymes are established in human populations, significant increase after an incidence can indicate the extent of blast injury in the absence of protective vests and aid in medical countermeasures. On the other hand, measurement of these enzymes in the plasma before deployment will address basal level variation of these enzymes in military population and will help to more precisely determine the severity of blast injury. Increase in the activities of plasma enzymes can also distinguish blast versus non-blast injuries or other types of trauma which are more localized.

## Materials and Methods

### Chemicals and reagents

Diagnostic kits to measure the activities of AST, ALT, LDH and CK in tissues were purchased from Randox Laboratories (Kearneysville, WV, USA). Phosphate buffered saline (PBS) and 4% paraformaldehyde (PFA) were purchased from Fisher Scientific (Pittsburgh, PA, USA).

### Animals and blast exposure

All animal experiments were conducted in accordance with the Animal Welfare Act and other federal statutes and regulations relating to animals and experiments involving animals and adhered to principles stated in the Guide for the Care and Use of Laboratory Animals (NRC Publication 1996 edition). The protocol was approved by Institutional Animal Care and Use Committee, Walter Reed Army Institute of Research. C57BL/6J male mice (8–10 weeks old) that weighed between 21–26 g (Jackson Laboratory, Bar harbor, ME, USA) were used in this study. Mice were housed at 20–22°C and 12 h light/dark cycle with free access to food and water *ad libitum*. A compressed air-driven shock tube was used for blast exposure of mice [12], [37]. Mice were exposed to single and repeated blasts as described earlier [37]. Briefly, mice were anesthetized with 4% isoflurane gas (O_2_ flow rate 1.5 L/min) for 8 min and quickly placed on a holder. The animals were restrained with a net to minimize the movements and any related injury during blast exposure. The holder was placed 2.5 feet inside from the open end of the 15 feet long shock tube and animals were placed in prone position, perpendicular to the direction of shock waves. Animals were exposed to different levels of single blast overpressure (10, 15 and 21 psi) by rupture of Mylar membranes of various thicknesses. For repeated blast exposures, mice were subjected to three consecutive blasts (21 psi) with 1–30 min intervals between blasts. The first two blasts were with 1 min intervals and the third blast exposure was at 30 min after the second blast. These exposures were similar to breachers exposure (8–10 kg trinitrotoluene, 10–12 times in a day or more in the night) (USAARL Report No. 2010-16, www.dtic.mil) or multiple blast exposures in the battlefield.

At the end of each time period, the animals were anesthetized and blood was collected by cardiac puncture into heparinized tubes. Plasma was separated and used for enzyme analyses. After blood collection, the animals were perfused transcardially using PBS to remove blood from tissues and liver and skeletal muscle were dissected for enzyme analyses. For histopathological analysis, separate groups of mice were exposed to blast and perfused transcardially with 4% PFA and post-fixed in 4% PFA after blood collection.

### Determination of the contribution of direct head exposure to blast on the plasma enzyme activity

To determine the contribution of direct head/brain blast exposure on the activity of enzymes in the plasma, the mice were protected with a snugly fitting vest covering the whole body except the head under anesthesia. Sham controls were treated in the same way except that the mice were not exposed to blast. The blood samples were collected after 6 h and subjected to enzyme analysis.

### Analysis of enzymes in the plasma

The activities of AST, ALT, LDH and CK in the plasma were determined using automated Vitros® 350 Chemistry System, Ortho Clinical Diagnostics (Rochester, NY, USA).

### Enzyme analyses in tissue homogenates

Activities of AST, ALT and LDH were determined in the liver, which is abundant source of these enzymes. CK is mostly present in the muscle and the activity was determined from the skeletal muscle. For enzyme analyses in the liver and skeletal muscle, PBS perfused tissues were homogenized with 1∶7 (wt/vol) tissue protein extraction reagent (Pierce Chemical Co, Rockford, IL, USA) containing protease inhibitor cocktail (Sigma-Aldrich, St. Louis, MO, USA). The homogenate was centrifuged at 5000×*g* for 10 min and collected the supernatant. The activities of AST, ALT, LDH and CK were determined in the supernatant by using the diagnostic kits according to manufacturer's instructions.

### Histopathology

Histopathology analysis of the liver and skeletal muscle of mice was performed at 6 and 24 h post-blast exposure. Tissues were collected after transcardially perfusing the animals with 4% PFA and processed by routine histology procedures. Tissue sections (5 µm) were stained with Hematoxylin and Eosin and evaluated using an Olympus Model AX80 (Olympus, Center Valley, PA, USA) inverted microscope and photos were taken using Olympus Model DP70 camera attached to the microscope.

### Statistical analysis

Results were expressed as mean ± SEM. Statistical analysis was carried out by Analysis of Variance (ANOVA). A *p* value<0.05 was considered significant.
